# Supplementary material for: Splice-Junction-Based Mapping of Alternative Isoforms in the Human Proteome
Source: Cell Rep. Author manuscript; Available in PMC 2020 Jan 15. (PMC6961840; doi:10.1016/j.celrep.2019.11.026)
Supplement: 2 [file NIHMS1546469-supplement-2.zip › DF1/PXD006675/LeftAtrium/LeftAtrium_3_TMEM245_ESSEAVDRGESAPTLSTSPSPSSPSPTSPSPTLGR.pdf]

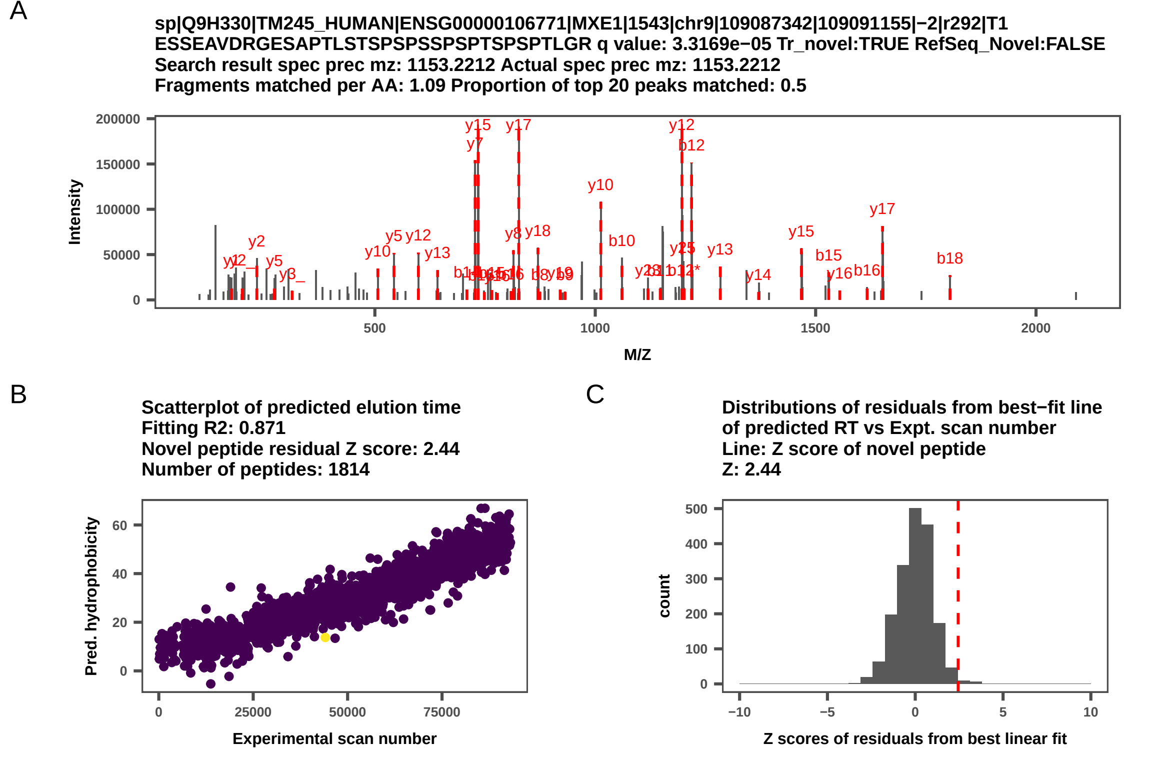
A
sp|Q9H330|TM245_HUMAN|ENSG00000106771|MXE1|1543|chr9|109087342|109091155|−2|r292|T1
ESSEAVDRGESAPTLSTSPSPSSPSPTSPSPTLGR q value: 3.3169e−05 Tr_novel:TRUE RefSeq_Novel:FALSE
Search result spec prec mz: 1153.2212 Actual spec prec mz: 1153.2212
Fragments matched per AA: 1.09 Proportion of top 20 peaks matched: 0.5
Intensity
200000
150000
100000
50000
0
500
1000
1500
2000
M/Z
B
Scatterplot of predicted elution time
Fitting R2: 0.871
Novel peptide residual Z score: 2.44
Number of peptides: 1814
Pred. hydrophobicity
60
40
20
0
0
25000
50000
75000
Experimental scan number
C
Distributions of residuals from best−fit line
of predicted RT vs Expt. scan number
Line: Z score of novel peptide
Z: 2.44
count
500
400
300
200
100
0
−10
−5
0
5
10
Z scores of residuals from best linear fit
